# A Computational Method to Assist the Diagnosis of Breast Disease Using Dynamic Thermography †

**DOI:** 10.3390/s20143866

**Published:** 2020-07-10

**Authors:** Thiago Alves Elias da Silva, Lincoln Faria da Silva, Débora Christina Muchaluat-Saade, Aura Conci

**Affiliations:** 1 Federal Institute of Piauí, Teresina 64000-040, Brazil; thiagoelias@ifpi.edu.br; 2 Institute of Computing, Fluminense Federal University, Niterói, Rio de Janeiro 24220-900, Brazil; debora@midiacom.uff.br (D.C.M.-S.); aconci@ic.uff.br (A.C.)

**Keywords:** breast disease, dynamic infrared thermography, cancer screening, tumor diagnosis

## Abstract

Breast cancer has been the second leading cause of cancer death among women. New techniques to enhance early diagnosis are very important to improve cure rates. This paper proposes and evaluates an image analysis method to automatically detect patients with breast benign and malignant changes (tumors). Such method explores the difference of Dynamic Infrared Thermography (DIT) patterns observed in patients’ skin. After obtaining the sequential DIT images of each patient, their temperature arrays are computed and new images in gray scale are generated. Then the regions of interest (ROIs) of those images are segmented and, from them, arrays of the ROI temperature are computed. Features are extracted from the arrays, such as the ones based on statistical, clustering, histogram comparison, fractal geometry, diversity indices and spatial statistics. Time series that are broken down into subsets of different cardinalities are generated from such features. Automatic feature selection methods are applied and used in the Support Vector Machine (SVM) classifier. In our tests, using a dataset of 68 images, 100% accuracy was achieved.

## 1. Introduction

Breast cancer is the most common type of cancer among women. The World Health Organization (WHO) states that 2.1 million women are impacted by this disease each year and it causes approximately 15% of all deaths among women [1]. Some factors related to breast cancer development are well known (aging, women’s reproductive life, family history, alcohol consumption, obesity, physical inactivity and exposure to ionizing radiation).

Due to its complex and multifactorial etiopathogenesis, up to date, there is no effective primary prevention for breast cancer [2], so efforts are directed to measures seeking secondary prevention. Early-stage disease survival can reach 98% in 5 years. Breast cancer screening aims at detecting asymptomatic tumors in order to reduce mortality from the disease and increase survival chances after diagnosis. Specific questions include determining who should be screened (risk stratification, the age to begin screening and to stop), and which method to use for screening.

Mammography is an examination considered as gold standard for cancer detection. However, even mammography has some limitations, such as high false positive rates, insufficient effectiveness in dense breasts and use of ionizing radiation. According to Arabi et al. [3], the risk of patient developing breast cancer increases 2% for each X-ray exposure. Moreover, for a better prognosis, minimizing the aggressiveness of the treatment and a decrease in mortality, early diagnosis assumes a decisive role.

**Figure 1:**
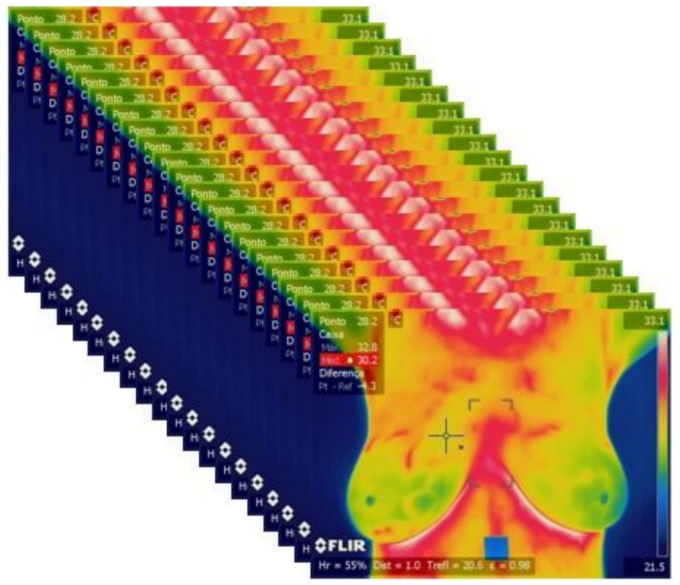
Capture of 20 sequential thermographies of a patient with a sick breast for 5 min by the used Dynamic Infrared Thermography (DIT) protocol.

Breast thermography could be another breast disease screening tool. It is based on the principle that breast tissues with some disease behave thermally different from healthy ones. Contrary to Figure 1, the temperature difference between healthy and diseased breasts is not always so evident. Often, normal temperature variation is quite subtle in the early stages of malignancy, and changes may go unnoticed by direct visual interpretation of thermographies. Normal breasts with increased vases are generally warmer and may be misinterpreted as abnormal. Therefore the subjective interpretation of thermographies often leads to false diagnoses and intelligent detection systems are required [4].

The main goal of this work is to define a set of efficient features to be used in the construction of a Support Vector Machine (SVM) classification model to classify patients having or not having breast abnormalities, using thermographies obtained by Dynamic Breast Thermography (DIT). The proposed method is based on time series derived from features extracted of breast DIT images. Initially the images are pre-processed and several features are computed. The best features are selected and used to build a classification model. This model was evaluated and achieved an accuracy of 100% in our tests. This paper is an extended version of the paper described in [5].

The remaining text is organized as follows. Section 2 presents the basic concepts involved in the problem addressed. Section 3 informs of the material used in our work. Section 4 describes the proposed method in detail. Section 5 presents the results achieved by the proposed method. Section 6 discusses the results and makes comparisons with related work. Section 7 describes the conclusions and future work that can be developed from the method and results here presented.

## 2. Basic Concepts

Thermography is the recording of the body’s temperature distribution using infrared radiation emitted by its surface. Special cameras are used to detect such radiation and, along with other parameters, they are used to compute the object temperature [6]. Advances in infrared camera technologies have increased the application of the system in the medical field [7]. Breast thermography has been considered a method to aid in the screening of breast cancer, because anatomical changes in tissue may be preceded by physiological changes such as an increase in metabolic activity [8].

The different thermal behavior of breasts with cancer can be explained due to the pathological angiogenesis process, which is the emergence of blood vessels for supplying the tumor [6]. In this process, there is a decrease in flat muscle cells of the vessels making them unable to perform the vasoconstriction that would normally occur [9]. Moreover, the nitric oxide is a vasodilating substance that is also locally produced by cancer cells; and the inflammation process caused by cancer [6]. Thermography has advantages in screening for breast disease in younger women, because mammography is not indicated for women with dense breasts [10]. Infrared thermography can be categorized in relation to the body’s behavior under heat transfer, and classified as static or dynamic.

### 2.1. Static Infrared Thermography

Static Infrared Thermography (SIT) is the measurement of the temperature distribution of a scene at a given time when all the elements are in thermal equilibrium in the environment. In breast SIT, the procedures for preparing the examination room and the patient must be strictly respected so that the generated thermograms are physiologically neutral and free of thermal artifacts, ready for interpretation and diagnosis. Such procedures make up the image acquisition protocol which is divided into the following parts: recommendations to the patient; examination room conditions; patient preparation; and parameters capture [11].

### 2.2. Dynamic Infrared Thermography

When compared to SIT, Dynamic Infrared Thermography (DIT) is faster and more robust. This is due to the fact that the SIT requires significantly long time for acclimatizing the patient to the conditions of the examination room. On the other hand, DIT is much less dependent on the conditions and temperature of the environment [12]. While SIT presents a static technique for observing the distribution of temperatures on the surface of the breasts, DIT monitors or quantitatively measures the temperature changes on that surface in a given period of time. Rapid changes in the temperature of human skin produce valuable physiological and pathophysiological information, that cannot be obtained by mapping static temperatures by SIT [13].

After a thermal stimulus, TID monitors the dynamic changes in skin temperature. The most simple used thermal stimulus is air directed to the breasts using an electric fan. Cooling the breasts by air, theoretically, improves the thermal contrast between healthy and diseased tissues in the captured images. This is because, as already mentioned, the blood vessels produced by cancerous tumors have practically no elements that prevent the flow in the opposite direction as normal veins and nerve endings like the embryological vessels. These vessels are just endothelial tubes and therefore do not respond normally to a sympathetic stimulus. Thus, the cancer region remains practically unchanged when the breast is cooled. Healthy regions of the breast show more reduction in temperature [14].

## 3. Materials

The images used in our work were obtained from the Database for Mastology Research with Infrared Image—DMR-IR, accessible at http://visual.ic.uff.br/dmi. This is intended for the storage and retrieval of information from breast exams and clinical data from volunteers whose images were acquired at the University Hospital of Federal Fluminense University in Niterói, Brazil. The use of such data and the research were approved by the Research Ethics Committee of the hospital and registered in “Plataforma Brasil” under the number of Certificate of Presentation for Ethical Assessment 01042812.0.0000.5243 of the Brazilian Ministry of Health.

The DIT acquisition protocol is described in Silva et al. [15]. For each patient, 20 thermographies are acquired by this DIT protocol. A total of 1280 images were used in the learning phase. Figure 1 shows the 20 sequential images captured from a patient.

There were 32 patients with any breast anomaly. Although the number of healthy patients was greater than 32, we used a balanced set for building and testing the classification model, that is, 32 of each class, where healthy patients were chosen randomly from the dataset. In this study, a patient without breast anomalies is the one who performed the screening mammography with findings classified as 1 or 2 BI-RADS (Breast Imaging Reporting and Data System) category.

On the other hand, a patient with a breast anomaly has breast cancer confirmed by biopsy or has undergone a screening mammogram and its examination was classified as BI-RADS category 0. Some examples of anomalies in our dataset are: fibroadenomas, nodules, papillary discharge of serous fluid, phyloid breast tumor, liquid lesion, florid ductal hyperplasia, residual carcinoma, papillary carcinoma, infiltrating ductal carcinoma and lobular carcinoma in situ.

In order to acquire the thermograms, a camera of the FLIR manufacturer, model SC620, was used [16], which has a sensitivity of less than $0.04{\;}^{\circ }C$ and a standard range from $-40{\;}^{\circ }C$ to $500{\;}^{\circ }C$. Acquired images have a dimension of 640 × 480 pixels. Table 1 summarizes the data and camera features used.

## 4. Method

**Figure 2:**
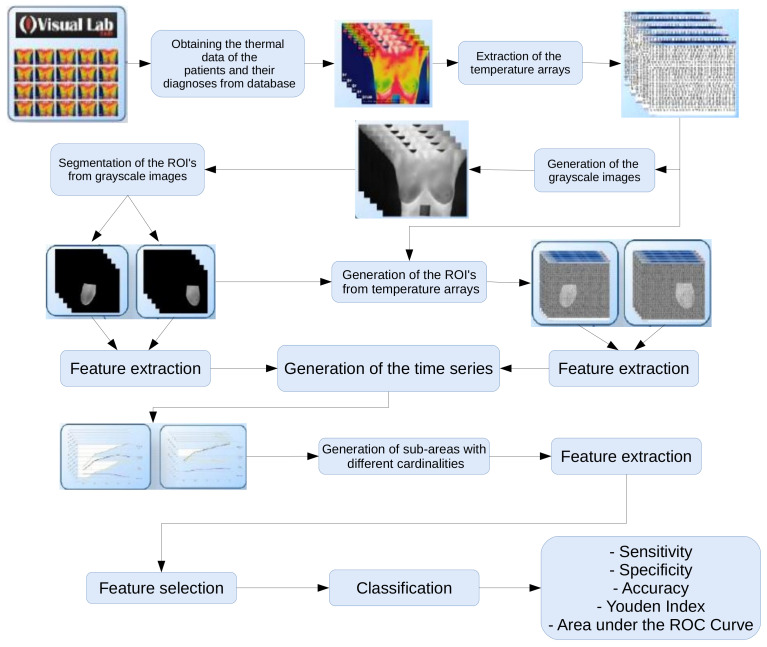
Flowchart of the proposed method.

Figure 2 represents the steps of the proposed method: (i) extraction of temperature arrays, (ii) generation of gray scale images from temperature arrays, (iii) segmentation of region of interest (ROI), (iv) feature extraction (v) feature selection, (vi) classification and (vii) validation of the results with performance metrics. The following sections explain each step in detail.

### 4.1. Extraction of Temperature Arrays

**Figure 3:**
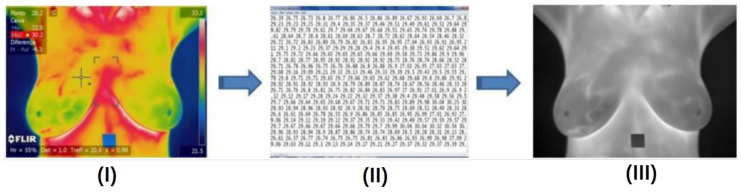
Extraction of temperature arrays from acquired frames and creation of an image from the temperature information. Copyright © 2019 IEEE, all rights reserved.

A temperature array is a text file with the temperature of each point in the scene captured by the camera. In such a file the temperatures are in degrees Celsius. For computing these arrays, the QuickReport application [17] was used, since the images generated by the camera have a proprietary format from the manufacturer, FLIR, called radiometric JPEG. The application receives a thermogram (image (I) in Figure 3) as input and can return the temperature array of that image (text file of image (II) in Figure 3) as an output. The computation of these arrays allows the use of some features based on the real temperatures (in Celsius degrees and in decimals) and not only the integer transformed gray scale values. The aim is to increase the precision of the information extracted from the breasts, since when quantifying the arrays for the generation of tonal images, detail of the information is lost.

### 4.2. Generation of Gray Scale Images

In order to compute features based on spatial relation among pixels, the temperature arrays extracted from patient sequential frames are used to generate gray tone images for each frame. Figure 3 illustrates it showing an image generated from the temperature array, image (III).

The generation of these images is done by two reasons. The first is the need to segment the region of interest that will be done in the next step of the method. The second reason is the computation of some image-based features. The segmentation of the region of interest is very important. It is necessary since the analyses performed in this work are based on the region of the patients’ breasts, disregarding the background of the image and the rest of the thorax. The analyses based on the temperature arrays will use the limits of the segmented breasts as a mask in order to consider only the temperature array values belonging to the regions of the breasts.

The gray scale images from the temperature arrays are defined according to Equation (1).

(1) pixelvalue[i]=(255·(pixelvalue[i]−min))/(max−min)

where *pixelvalue[i]* is the pixel value of the image and *min* and *max* correspond to the lowest and highest temperature values of the array. Each temperature value of the array is used in the transformation to obtain the corresponding pixel of the gray scale image. It is noteworthy that during the process of generating images in gray scale, the same maximum and the same minimum values of the temperature arrays were used for all exams. In other words, the highest and lowest temperature of all the temperature arrays used in the study were first of all obtained. Thus, it was ensured that all the images were generated using the same scale and a comparison could be made among them.

### 4.3. Segmentation of Regions of Interest

**Figure 4:**
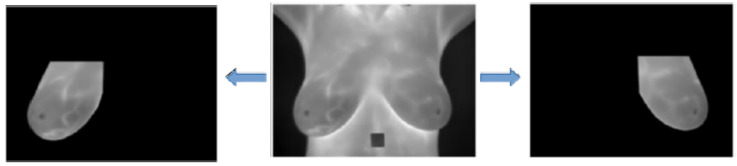
Result of the Region of Interest (ROI) segmentation. Copyright © 2019 IEEE, all rights reserved.

The DIT thermal images contain areas of the patient’s skin that are not important in the context of the addressed problem: only the breasts are considered to be the Region of Interest (ROI). The segmentation of the ROI for a given patient can be seen in Figure 4.

All 1280 images (64 breasts, each with 20 sequential images) used in the learning stage of the method and the 240 images (12 breasts, each with 20 sequential images) used in the test step were manually segmented using the GIMP tool [18], which is a free image editing software. During this process, three people were responsible for these segmentations. Two of them were responsible for manual segmentation, while the third checked whether segmentation was done properly. If it was not proper, segmentation was repeated. All segmented images are available at http://visual.ic.uff.br/proeng/thiagoelias/.

**Figure 5:**
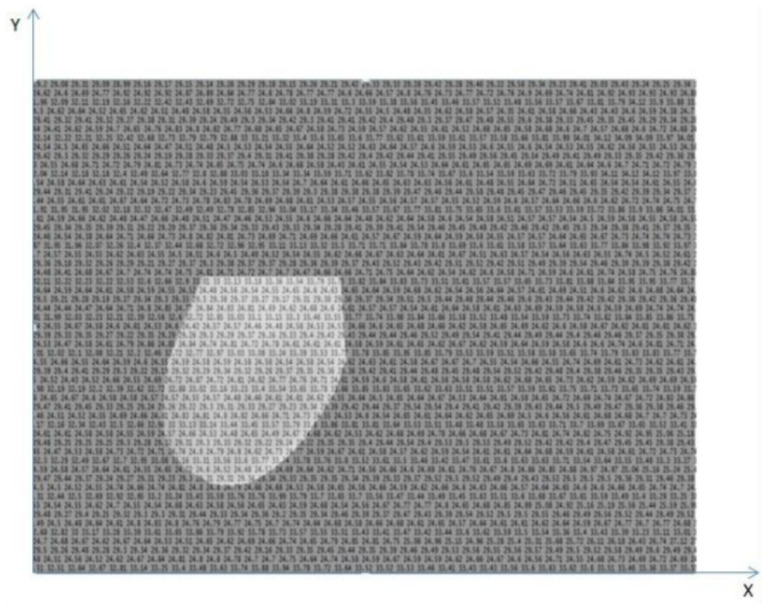
Determination of the region of interest (ROI) of the temperature array from the ROI in gray scale.

Even during the extraction process of the features based on the temperature arrays (not on the images in gray scale), it is necessary to use the segmented images in gray scale, because such images serve as a mask for the arrays. Thus, only the temperature elements belonging to the breasts are considered in the computation of the features. Figure 5 exemplifies the use of ROI in gray scale for determining the temperature arrays inside the ROI.

### 4.4. Extraction of Features from the Regions of Interest

**Figure 6:**
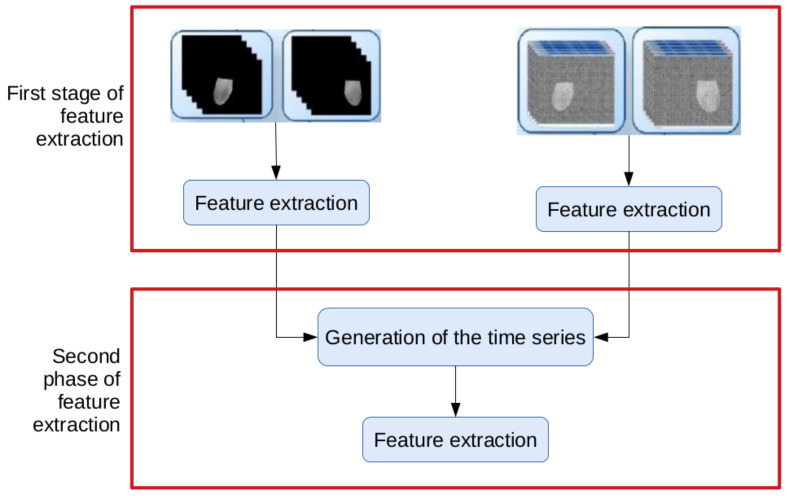
The two phases of the feature extraction.

The extraction of features is performed in two phases, as illustrated in Figure 6. In the first phase, features are extracted from the ROIs of the gray scale images and the temperature arrays and used to form time series. In the second phase, features are extracted from the time series: they are latter features that will be used in the classifier to discriminate the patient as healthy or ill.

#### 4.4.1. Extraction of Features: First Phase

**Figure 7:**
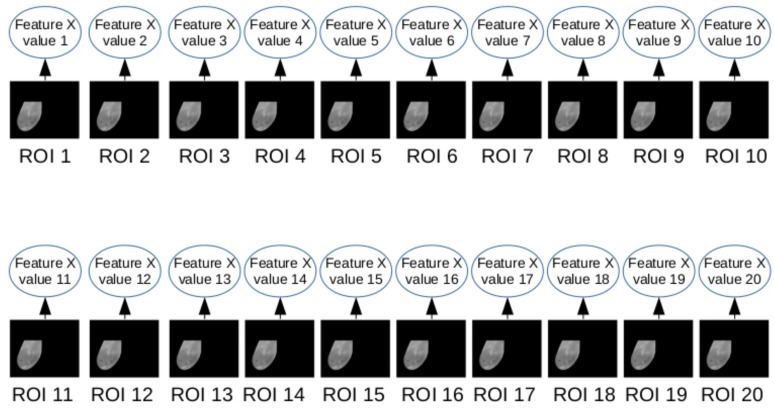
Representation of features based on an individual ROI.

**Figure 8:**
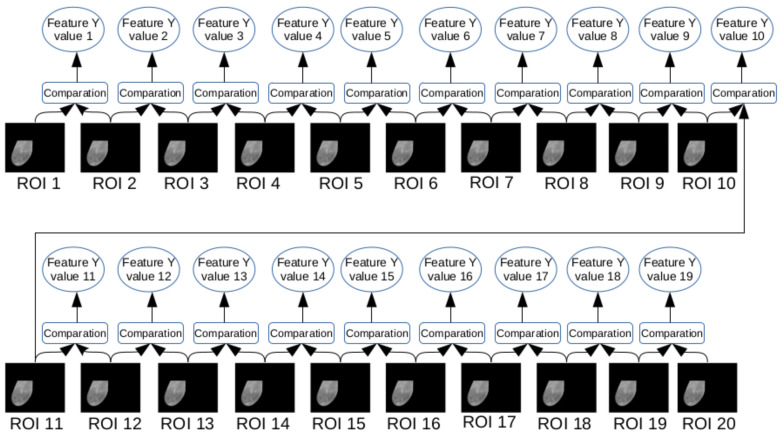
Representation of features based on comparison of consecutive ROIs.

In this phase, features are computed from the gray scale images and temperature arrays (both generated from the infrared data obtained by DIT exams). Each breast, as explained in the previous sections, has Q(i) images and arrays, where *i* varies from 1 to 20. Therefore, the features were extracted from each of the ROIs, for some features, where 20 values were obtained, one value for each frame *i*. Figure 7 exemplifies the case where the feature depends on a single ROI. For features based on comparison of two ROIs, 19 values were obtained. Figure 8 illustrates the case where the feature depends on two consecutive ROIs: ROI *i* and ROI i+1. The values of all features serve as a basis for the next phase of the features computation.

##### 4.4.1.1. Features Based on Simple Statistics

These features are the *Average Temperature* and the *Standard Deviation*. They are extracted from the ROI’s temperature values, i.e., considering only the temperatures of the breast regions of the patients. For this purpose, the segmented images of the breasts were used as mask for the temperature arrays, as detailed in Section 4.3. For each of the 20 sequential arrays of a breast on analysis, the average temperature and standard deviation were calculated, generating 20 values for each of these features.

##### 4.4.1.2. Cluster-Based Features

Cluster-based features are also computed from the temperature arrays, and do not from gray scale images. The temperatures in Celsius degrees of the sequential images of each breast are analyzed. The segmented images of the breasts are used as masks for the arrays. To extract these, the *K*-means clustering algorithm proposed by MacQueen [19] was used. The amounts of 3, 6, 9, 12 and 15 seeds were tested, but the best results were obtained with 9 seeds. The choice of the 9 seeds for the initialization of the algorithm was based on the proposal of Arthur and Vassilvitskii [20], which consists of an approximation algorithm for the *k*-means NP-hard problem. After completing this step, each element of a given group was re-positioned in another group in order to verify if this new positioning generated a better degree of internal homogeneity than the group generated randomly. The degree of homogeneity was obtained from the sum of residual squares, obtained by Equation (2).

(2) $$SQRes\left(j\right)=\sum_{i=1}^{n_{j}}d^{2}\left(o_{i}\left(j\right);\bar{o}\left(j\right)\right)$$

where $o_{i}\left(j\right),\bar{o}\left(j\right)$ and $n_{j}$ are respectively the values of the *i*-th register, the center of group *j* and the number of records of this group. The movement of elements between groups to try to obtain the greatest internal homogeneity happened 1000 times with an accuracy of 0.01. After the grouping stage of each breast temperature array, the centroid with the highest temperature value among the 9 centroids was identified. This value of the largest centroid was used as a feature and that feature received the name of *Grouping*. This procedure was repeated for the 20 sequential arrays of each breasts.

##### 4.4.1.3. Features Based on Comparison of Histograms

An image histogram is a quantification of the number of pixels for each image gray level. In other words, it is a representation of the intensities presented in an image [21]. Here, the comparison of two histograms are done based on Bhattacharyya Distance, Chi-square Distance and Intersection Distance [22]. We call these features, respectively, by *Battacharyya*, *Chi-square* and *Intersection*. In this work, the comparison is made between two subsequent frames, that is, the distances between *i*-th histogram and (i+1)-th histogram. Thus, as two histograms are necessary to generate a single value, for each feature based on histogram comparison of a given breast, 19, i.e., (20−1), values were obtained.

##### 4.4.1.4. Features of Texture from the Histograms of Sum and Differences

Six features presented by Baraldi and Parmiggiani [23] were analyzed from the sum and difference histograms (Section 4.4.1.3), as proposed by Unser [24], for the study of the texture of each breast. These features are: energy, entropy, contrast, variance, correlation and homogeneity. The analysis was carried out in two ways: the first considering the normalized histograms of the sum and difference with Dx=1 and Dy=0, generating the features that we will call *Horizontal Energy*, *Horizontal Entropy*, *Horizontal Contrast*, *Horizontal Variance*, *Horizontal Correlation* and *Horizontal Homogeneity*; and the second considering sum and difference of normalized histograms with Dx=0 and Dy=1, generating the features that we will call *Horizontal Energy*, *Vertical Entropy*, *Vertical Contrast*, *Vertical Variance*, *Vertical Correlation* and *Vertical Homogeneity*.

##### 4.4.1.5. Features of Texture from Diversity Indexes

The following ecological diversity indexes were used to analyze the 20 sequential images of each breast in this work: Macintosh, Simpson and Berger-Parker index [25]. The calculation of these indexes was performed on sum and difference histograms. The population considered was all frequencies of sum and difference histograms (Section 4.4.1.3). The species were created by segmenting the sum and difference histograms into groups of 7 bins. For example, considering the histogram of sums, which ranges from 0 to 510, the first species is considered from bin 0 to bin 6; from bin 7 to bin 13 it was considered the second species and so on. The same was done with the histogram of differences, which ranges from −255 to 255. In total, 146 different species were created (73 generated from the histogram of sums and 73 generated from the histogram of differences).

As in the last subsection, because these features are also based on sum and difference histograms, two neighborhoods on computing these histograms are considered. The histograms of sum and difference with neighborhood Dx=1 and Dy=0 and with neighborhood Dx=0 and Dy=1. Thus, six features were generated that we will call *Horizontal Macintosh*, *Horizontal Simpson*, *Horizontal Berger-Parker*, *Vertical Macintosh*, *Vertical Simpson* and *Vertical Berger-Parker*.

##### 4.4.1.6. Features Based on Spatial Statistics

Like histograms, spatiograms also capture high-order moments of an array or image. The second order moment’s of spatiogram of an object is similar to a histogram of same object, except that it also stores additional spatial information. Here, the mean and covariance considering the spatial position of pixels falling on each bin of the histogram is used [26]. As in the comparison of histograms, the spatiogram comparisons follow the sequential order of frames in the examination: the i-th frame was compared to the spatiogram of the (*i* + 1)-th frame. Thus, at the end, 19 spatiograms were compared for each breast. However, in order to perform correct positional comparison, the sequential images of each breast must be submitted to the process of image registration. The technique used in this work for registration was presented in [27]. This feature will be called *Spatiogram*.

##### 4.4.1.7. Features Based on Fractal Geometry

The fractal dimension can be seen as a measure of the irregularity of many physical processes. However, different fractal sets with diverse textures may share the same fractal dimension value [28]. To discriminate these textures, fractal dimension alone would be useless. Thus, Mandelbrot [28] introduced a second measure: the lacunarity to better distinguish fractals of the same dimension but with different texture appearances. Although there are several authors that propose different methods to calculate the lacunarity, we chose to use the method proposed by Du and Yeo [29], using the images in gray scale. The reason why we chose to use gray scale images instead of binary images, which have a lower computational cost, is because the process of binarization causes loss of information, reducing the study possibilities. In this work, for computation of the *Lacunarity* feature, the following sizes were used in sliding window pixels: 2 × 2, 4 × 4, 8 × 8, 16 × 16, 32 × 32 and 64 × 64.

#### 4.4.2. Extraction of Features: Second Phase

The second phase corresponds to the computation of features of the time series generated from the values of the features extracted in the previous phase. For each feature calculated from the sequential ROIs of the gray scale images or from the sequential ROIs of the temperature arrays of a given breast, a corresponding time series was generated. Then, from these series, new features were extracted to be submitted to the classifier.

**Figure 9:**
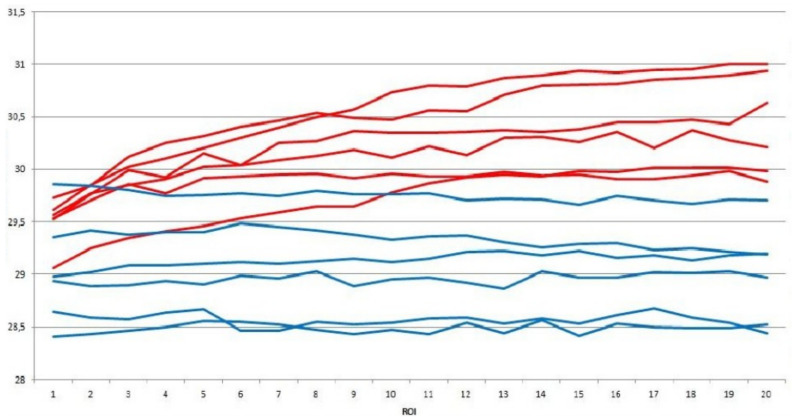
Time series of average temperature for sick (red) and healthy (blue) breasts.

Figure 9 shows an example of the time series, where on the horizontal axis there are the sequence of the 20 ROIs from DIT exams and, on the vertical axis there are the average temperature values of these for some breasts. Red lines were used to represent the series of breasts with diagnosis of cancer and lines in blue represent the series of healthy breasts.

In the next two sections, the process of generation of the time series with their different number of elements (cardinality) will be presented, as well as the process of computation the various types of features.

##### 4.4.2.1. Creation of the Time Series

A time series is a sequence of data obtained at regular intervals of time during a specific period. In the analysis of a time series, the main desire is to model the studied phenomenon to describe the behavior over time [30]. Here, given a breast and considering a particular feature extracted from its N gray scale images or temperature arrays, a time series is obtained when these N values are ordered representing the variation of the feature over time. This is done while the recovery of the breast temperature occurs, after the cooling is induced, as already described.

Based on the Higuchi proposal [31], from a time series, sub-series of different resolutions can be generated and also analyzed for better observation of a behavior at a later stage. Given a one-dimensional series of *N* values X=x(1),x(2),…,x(N), new series are generated by Equation (3):

(3) $$X_{k}^{m}=x\left(m\right),x\left(m+k\right),x\left(m+2k\right),\ldots ,x\left(m+int\left(\frac{N-m}{k}\right)k\right)$$

where *k* and *m* are integers: *k* represents the discrete interval between the points and *m* indicates the initial value of the analyzed series with m=(0,1,…,(k−1)). The number of elements of each new series generated is calculated using Equation (4):

(4) $$L\left(m,k\right)=\left\{\frac{\sum_{i=1}^{\left\lfloor \frac{N-m}{k}\right\rfloor }\left|x\left(m+ik\right)-x\left(m+\left(i-1\right)k\right)\right|\left(N-1\right)}{\left(\lfloor \frac{N-m}{k}\rfloor \right)k}\right\}/k$$

where ⌊(a)⌋ represents the integer part of (a), (N−1)/⌊(N−m)/k⌋k is the normalization factor of the series. In this work, the value of *k* ranges from 1 to 4. Thus, 10 sub-series can be generated for each feature proposed, as can be observed in Table 2.

**Figure 10:**
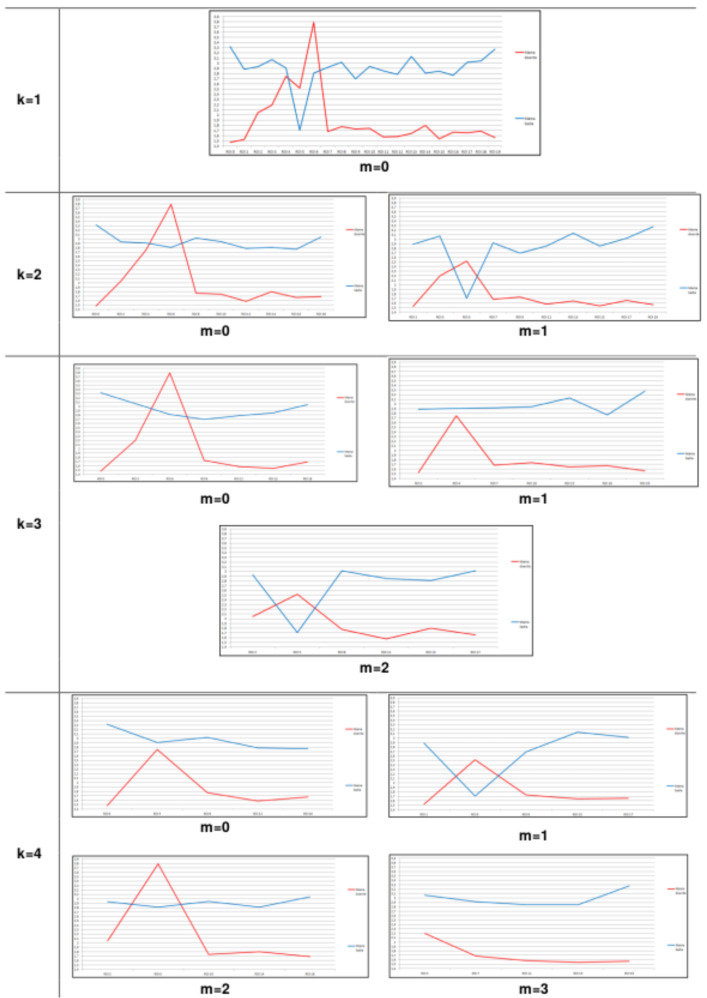
Time series of two breasts, healthy (blue) and sick (red), with different cardinalities.

Figure 10 shows an example of the 10 resolutions generated from the sub-series of a given feature for two breasts, varying the *k* and *m*, as explained previously. In the horizontal axis, the sequential images are represented and, in the vertical axis, the value of the feature is shown. The series in red represents the feature extracted from the sequential images of a sick breast. The series in blue represents the same feature extracted from images of a healthy breast. The *k* and *m* present different values when generating sub-series in new resolutions. The main goal of new series is to eliminate outliers that may arise, as observed in the first one of Figure 10 ( k=1,m=0). In small time increments, there is no clear separation between sick and healthy breasts. On the other hand, it is possible to note that, when k=4, m=3, the separation between the sick breast and healthy breast is clear. Same procedures were repeated for all features computed from each breast analyzed in this work.

##### 4.4.2.2. Extraction of Features of Time Series

**Figure 11:**
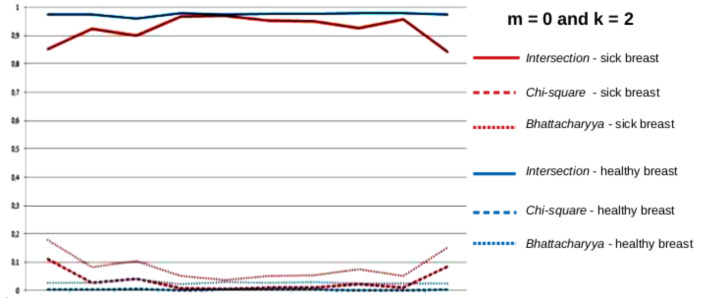
Time series based on the comparison of histograms, for healthy (blue) and sick (red) breasts, k=2 and m=0.

**Figure 12:**
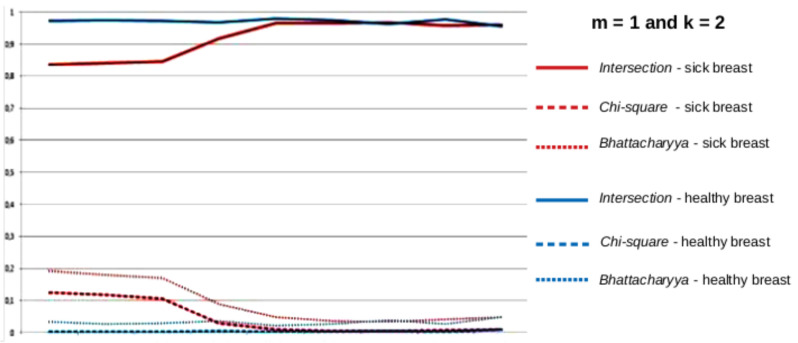
Time series based on the comparison of histograms, for healthy (blue) and sick (red) breasts, k=2 and m=1.

After the time series of all the features of each breast were created, new features are computed from them. Firstly, the series were separated by the groups of features described in Section 4.4.1. Secondly, each group was subdivided by the type of the series, i.e., from the values of *k* and *m*. Therefore groups of series were formed as, for instance, those in Figure 11 and Figure 12. These figures show the series (with k=2) of the group of features based on the comparison of histograms, explained in Section 4.4.1, for two breasts: one healthy and one sick in blue and red, respectively. The group of features is formed by three distances between histograms: *Battacharyya*, *Chi-square* and *Intersection*. Thereby, for each breast, there are 3 time series representing each of the 3 features of each group. The series represented by the solid line represents the intersection between the histograms, the dashed line represents the *Chi-square* distance of the histograms and the dotted lines represent the *Bhattacharyya* distance of the histograms.

After the organization of the time series by group of features and by the type of the series, the second phase of the feature extraction process started, which corresponds to the computation of features from these new time series. Now for each time series, two new features were computed. The first corresponds to the range (R) of the features, that is, the difference between the largest and the smallest value of the characteristic described in the time series. The second feature corresponds to the square root of the sum of the square of the time series values (M2). They are defined by Equations (5) and (6), respectively.

(5) $$R=Max\left(x_{i}\right)-Min\left(x_{i}\right)$$

(6) $$M2=\sqrt{\frac{\sum_{i=1}^{n}x_{i}^{2}}{n}}$$

where $x_{i}$ represents the time series values and *n* is the number of values. Thus, using, for example, the series of dimensions k=2, m=0 of the group of features based on the comparison of the histograms, shown in Figure 11, for each breast, a total of 6 features was obtained: two features (range and square root of the variance) of each of the time series representing each one of the 3 comparisons of histograms.

### 4.5. Feature Selection

Sometimes, the feature extraction process generates redundancies or irrelevant features. This can compromise the performance of the algorithms in the construction of prediction models. Thus, it is necessary to apply techniques for selecting features, improving algorithm performance. Then a stage of selection of features was inserted in the proposed method aiming at the efficiency of the classifier. In other words, the goal of this step is decreasing the number of features to be used, finding a subset that is related to a correct classification, but with little correlation among them. In this work, the selection of features was performed in WEKA (Waikato Environment for Knowledge Analysis) tool using the CfsSubsetEva attribute evaluator [32] and the BestFirst research method.

### 4.6. Classification

The following step is to classify the breasts into sick or healthy (patients), therefore, the features extracted from the time series are considered elements of a vector representing the corresponding breast. The formed vector is submitted to the Support Vector Machine (SVM) classifier implemented in WEKA [32] to identify if it is sick or healthy after a learning process. Before classification, each vector has its elements normalized between 0 and 1 using Equation (7).

(7) $$Y_{i}=\frac{X_{i}-X_{min}}{X_{max}-X_{min}}$$

where $Y_{i}$ is the value of the attribute $X_{i}$ after normalization between 0 and 1, $X_{min}$ and $X_{max}$ are the minimum and maximum values respectively obtained inspecting the whole sample of the same group. The normalization is used to avoid that attributes with larger numerical ranges dominate those in smaller numerical intervals. It also avoids numerical difficulties during calculation because some processes generally depend on the internal products of feature vectors. Consequently, it is always recommended to normalize the attributes to [−1,1] or [0,1].

The used Support Vector Classification (SVC) allows classification in two classes. In order to generate the classification model, learning was configured in two ways: C-SVC and Nu-SVC. Where *c* is a regularization factor for the feature space and *n* is an upper limit on the fraction of training errors and a lower bound of the support vector fraction. The core function used for the classification was the Radial Basis Function (RBF), as indicated by Hsu et al. [33], and tests performed confirm that this function actually presented the best results. The leave-one-out validation technique was used to validate the classifier performance. It is a validation technique used to ensure that the results presented by a classifier are independent of the used dataset. The technique uses a single feature vector of a class as the validation data, and all remaining vectors of the sample, as the training data. This is repeated so that each feature vector in the sample is used once as validation data.

### 4.7. Results Evaluation

In order to evaluate the method proposed in this work, some metrics were calculated from the obtained results. They are: sensitivity, specificity, accuracy, Youden’s index and the area under the received operation characteristics (ROC) curve. Such measures were adopted since the great majority of the diagnostic systems also use them to demonstrate their results quality.

## 5. Results

In this section, the results achieved from the method proposed in the previous section are presented and discussed. A summary is presented first with the best classification results by group of features. Then, the feature selection is performed and the results are presented for each series. In the following subsection, the results of the classification based on the result of the feature selection are presented. Then, the results achieved are discussed and comparisons of the results achieved with the proposed method are also made with other related studies of the literature for the diagnosis of breast diseases based on infrared images.

### 5.1. Results by Feature Groups

**Figure 13:**
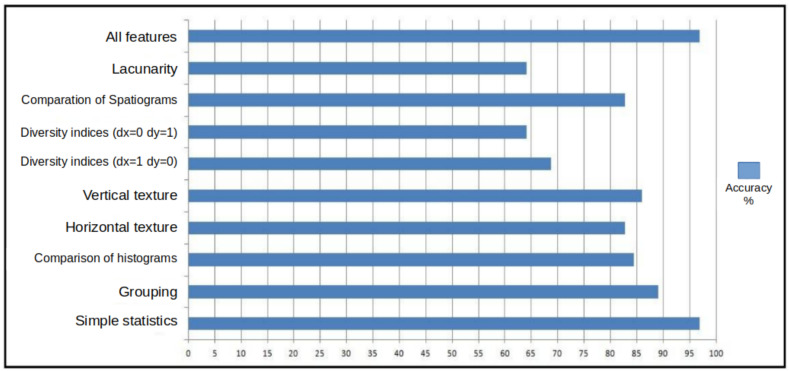
Highest accuracy by feature group.

For each group of features, the series were divided by types, as explained in the previous section. Two features that were submitted to the classifier were extracted from each series, they are: the amplitude of the series and the square root of the second moment (Section 4.4.2). The best results of the analyses explained above are shown in Figure 13. A new group formed by the union of all other groups was also considered.

Figure 13 shows on the vertical axis the groups of features considered in Section 4.4.1, and also a group formed by all features. On the horizontal axis, a scale is presented with the values as a percentage of the accuracy. The bars in the graph represent the highest accuracy values achieved by varying the size of the series and the classifier (C-SVC and Nu-SVC) for each group of features. Still in relation to Figure 13, it is possible to notice that the greatest accuracy was obtained by the features of the group of simple statistics and by the union of all features, reaching values close to 97% accuracy. On the other hand, groups formed by diversity indexes (horizontal and vertical) and lacunarity obtained lowest rates of accuracy.

### 5.2. Feature Selection

After the classification using the groups of features, the feature selection was considered aiming at selecting the most relevant features for classification. The number and names of the selected as best features for each time series can be seen in Table 3, where *R* represents the square root of the second moment and *A* represents the amplitude of the series.

From the results presented in Table 3, it can be seen that some features were not selected in any of the types of time series considered in this work. All features related to diversity indexes; standard deviation; the comparison of histograms using the chi-square; the horizontal variance; the horizontal correlation; the vertical variance; the vertical energy; the vertical correlation; the vertical entropy; and the vertical homogeneity were not selected for any time series. On the other hand, other features such as those related to the *Average Temperature*, clustering, comparison of histograms using the intersection, horizontal energy, vertical contrast and lacunarity were selected in all or most of them.

### 5.3. Classification with Selected Features

After the feature selection step, the classification process was redone considering only the features selected in the previous step for each of the time series considered. The results can be seen in Table 4 and Table 5. As in the previous classifications, all SVM parameters were default, varying only between C-SVC and Nu-SVC. In Table 4, the line highlighted in blue contains the set of features recommended in this work to distinguish healthy patients from those with breast abnormalities. Such a recommendation is detailed in Section 6.

## 6. Discussion

The best results were achieved after the feature selection, obtaining an accuracy of 100% in 5 different time series with their respective selected features, as shown in Table 4 and Table 5. For the choice of the series recommended by this work and its respective selected features, the lowest number of ROIs and the smallest number of selected features were adopted as selection criteria.

Although 100% accuracy has been achieved in 2 different types of time series when using C-SVC and in 3 types of series when using Nu-SVC, only the series of k=4 and m=0 using C-SVC, with their respective selected features, is recommended by this work for the construction of the classification model.

Analyzing the 5 types of time series and their respective selected features, which provided 100% accuracy, in two of them (k=4, m=2 with C-SVC and Nu-SVC) 7 features were selected. These are not recommended by this study since they require a greater number of features extracted from the sequential ROIs of each breast.

Considering the series with k=2 and m=0 using Nu-SVC, despite reaching 100% accuracy by selecting 6 features, as it is a time series of k = 2, this is formed by a larger number of ROIs. To obtain these series, it was necessary to extract the 6 features from 10 sequential ROIs.

Therefore, only the series with k=4, m=0 considering C-SVC (Table 4) and k=4, m=3 considering Nu-SVC (Table 5) remained. In both, the time series were constructed from the features of 5 sequential ROIs for each breast. Additionally in both, for the construction of the series, only 6 features were extracted. However, comparing the features of the two series, it is possible to conclude that the series with k=4 and m=3 requires the construction of the histograms of each ROI to calculate the *R Intersection* and *A Vertical Contrast* features, while the series with k=4 and m=0 do not need the additional calculation of such histograms to calculate the *R Horizontal Energy* and *A Horizontal Energy* features, thus demanding less computational effort. The other features of the two series are the same.

Thus, we recommend to build the classification model using the features of the series k=4, m=0, considering all the default parameters of the SVM implemented in the WEKA tool, including the default variable C-SVC for learning. The six recommended features to be extracted from the time series formed by the features of the ROIs of the gray scale images or the ROIs of the temperature matrices are highlighted in blue int the Table 4.

With this recommendation, the proposed method achieved 100% accuracy using the SVM classifier with all its default parameters and the leave-one-out cross-validation technique. It is worth mentioning that these results are valid for the database used in the development of the method. However, they demonstrate an estimate of the potential of the method proposed in the task of classifying breasts that do not belong to the considered base.

Thus, the objective of this work was achieved, which was to determine a set of efficient features (highlighted in blue in the Table 4) for the construction of a classification model for the task of classifying patients as healthy or with breast anomalies, based on DIT.

### Related Work

Although there are many studies using infrared images for breast cancer, the following paragraphs consider only those using DIT technique for screening, which are more related to our work.

Anbar et al. [34] used a camera for image acquisition with a rate of 100 images per second. The breasts are cooled by an electric fan. Only patients with suspected mammograms, which justify the biopsy, were included in the tests. After the acquisition of the thermograms, these patients underwent surgical intervention and were classified according to the disease: patients with invasive cancer, patients with ductal carcinoma in situ and patients with benign lesions. The region of interest, in this case the sick breast, is divided into small regions of size 4 × 4 pixels. The time series of a given region is constituted by the average temperature value, of that region, in the 1024 images of the thermogram sequence. The Fast Fourier Transform is applied over each time series generating an energy spectrum, for each one. Features are extracted from these spectra and statistical analyses are performed on them in order to discriminate between healthy and sick breasts. The conclusions are based only on graphs and tables, without using computational techniques in decisions. Specificity and sensitivity above 95% were achieved in some tests, using 100 patients, 343 with cancer and 66 with benign tumors.

In the work developed by Wishart et al. [35], images are obtained by DIT. For 5 min, a cold air flow was directed to the patients’ breasts and during this period 250 breast thermographies are acquired by a thermal camera. Images of 106 patients were used. From that group of patients, 65 are diagnosed with cancer and 41 with benign lesions. Thermal asymmetry features and temperature differences were extracted from the sequential images. The authors did not determine the features that were extracted from the images. For classification, an artificial intelligence method developed by the authors called NoTouch BreastScan was used. The authors did not clarify how many features were exactly extracted nor did they detail the classification technique. The method obtained a sensitivity of 70% and specificity of 48%.

Gerasimova et al. [36] proposed a multifractal analysis of DIT as an efficient method to identify women at risk of breast cancer. They used a Wavelet-based multiscale method to analyze temporal fluctuations in breast skin temperature obtained from a group of breast cancer patients and some healthy breast volunteers. The authors demonstrated that the multifractal complexity of temperature fluctuations observed in healthy breasts is lost in breasts with malignant tumors. Forty-seven patients participated in the study, being 33 diagnosed with cancer and 14 healthy. The classification was performed visually through graphs and tables. The authors obtained a sensitivity of 76% and specificity of 86% and used a camera for image acquisition with a rate of 50 images per second.

Saniei et al. [37] developed a method where only two images are used. The patient is instructed to immerse her hands in a container with water and ice for 1 min (temperature of approximately $5{\;}^{\circ }C$) in order for vasoconstriction to occur on the breast surface. Immediately after removing the hands from inside this container, another thermogram is captured. By computational techniques, both breasts are segmented and images are recorded. The next step is the segmentation and skeletonization of the vascular pattern present in the breasts, in two thermograms. Features are extracted from these vascular patterns and techniques similar to those used for fingerprint recognition are applied to compare vascular patterns, from thermograms before and after thermal stress. In order to quantify the degree of similarity between the patterns in the two images, a relationship score is generated by means of a mathematical expression and these patterns will be considered if the score is below a certain threshold, which is defined empirically. According to the authors, the method reached a sensitivity of 86% and specificity of 61%. Images of 50 patients, 25 with and 25 without breast cancer, were used.

Silva et al. [15] developed a method of analyzing thermal images of the breast obtained by DIT. In this method, the patient is classified as healthy or having an anomaly in the breast. Time series of temperature were generated from the region of interest (the breasts) and unsupervised and supervised machine learning techniques were applied to them. A database with 70 patients was used, 35 healthy and 35 with some breast anomaly. According to the authors, the method reached a sensitivity of 98% and specificity of 100%. A camera with a rate of 0.066 images per second was used for image acquisition.

Baffa and Lattari [38] used deep learning (a Convolutional Neural Network) to classify patients into healthy and unhealthy, using the thermographies obtained by DIT and SIT, from the DMR-IR database [39]. To analyze the images obtained by DIT, they used data of 137 patients, where 95 patients were considered healthy and 42 were assigned to the unhealthy set. In addition to assessing the correctness rate of the classification, the training time of the neural network was recorded. For the images obtained by DIT, four strategies were defined to generate a single image that would feed the neural network, for example, for each patient, to generate an image of the difference between the last and the first image in the image sequence. They obtained 98% of average accuracy with images obtained by DIT and 95% with images obtained by SIT.

Table 6 contains the main features of the studies presented in this section. Their proposed methods for systems to aid medical diagnosis, with the exception of Silva et al. [15] and of Baffa and Lattari [38], achieved decisions based on computationally processed graphics or images but did not use machine learning techniques, like our work does.

## 7. Conclusions

Breast cancer is the cancer that most affects women worldwide. The diagnosis of the disease in the first years is decisive for its cure. The main contribution of this work is the definition of a set of efficient features for the construction of the classification model to classify patients as healthy or with some breast anomaly using Dynamic Infrared Thermography (DIT).

The best results of this approach reached an accuracy of 100% using SVM implemented in WEKA with all its default parameters. The validation technique used was leave-one-out cross-validation. It is worth mentioning that these results were achieved using the Database for Mastology Research with Infrared Image—DMR-IR, accessible at http://visual.ic.uff.br/dmi. Therefore, future work would do the association of analysis of images and clinical data of patients to assist in the diagnosis of breast diseases. The proposed method is able to assist in the diagnosis of breast diseases, giving indications of which patient’s breast has an anomaly. However, it is not possible to indicate in which region of the breast the anomaly is located. Thus, it is suggested as a future work the development of techniques that, in addition to assisting in the indication of the possibly sick breast, indicate the region of the breast where the anomaly is located. Due to the effort required for manual segmentation of ROIs of the images to be used by the presented method, the development of an automatic technique for segmenting the ROIs is an important point.

## Figures and Tables

**Table 1:** Summary of data and camera characteristics used.

Total number of selected patients	64
Total number of healthy patients	32
Total number of patients with breast anomalies	32
Number of images per patient	20
Total number of images used	1280
Camera used in the acquisition of thermograms	FLIR model SC620 [16]
Thermal sensitivity	<0.04 ${}^{\circ }C$
Standard capture range	From $-40{\;}^{\circ }C$ to $500{\;}^{\circ }C$
Image resolution	640 × 480 pixels

**Table 2:** The cardinality of the time series generated for each feature.

**Serie**	1	2	3	4	5	6	7	8	9	10
**k value**	1	2	2	3	3	3	4	4	4	4
**m value**	0	0	1	0	1	2	0	1	2	3

**Table 3:** Selected features for each series type.

k	m	Feature	Names of
Value	Value	#	Selected Features
1	0	7	*R Average Temperature (RAT)*; *A Average Temperature (AAT)*; *R Grouping (RG)*;
			*R Horizontal Energy (RHE)*; *R Spatiogram (RS)*;
			*A Horizontal Contrast (AHC)*; *A Vertical Contrast (AVC)*
2	0	6	*RAT*; *AAT*; *RG*; *R Lacunarity (RL)*; *A Intersection (AI)*; *AVC*
2	1	8	*RAT*; *AAT*; *RG*; *A Horizontal Energy (AHE)*; *RHE*; *RS*; *AI*; *AVC*
3	0	7	*RAT*; *AAT*; *RG*; *RHE*; *R Horizontal Contrast (RHC)*; *RL*; *AVC*
3	1	5	*RAT*; *AAT*; *RL*; *AI*; *AHE*
3	2	7	*RAT*; *AAT*; *RL*; *RS*; *AI*; *A Horizontal Homogeneity (AHH)*; *AVC*
4	0	6	*RAT*; *AAT*; *RG*; *RHE*; *AHE*; *RL*
4	1	6	*RAT*; *AAT*; *R Horizontal Entropy (RHEtp)*; *RS*; *RL*; *AI*
4	2	7	*RAT*; *AAT*; *RG*; *RHEtp*; *RL*; *A Bhattacharyya (AB)*; *AVC*
4	3	6	*RAT*; *AAT*; *RG*; *AI*; *RL*; *AVC*

**Table 4:** Result of the classification with C-Support Vector Classification (SVC) for each series after the feature selection.

k	m	Sensit.	Specif.	Accur.	Youd.	ROC	#	Selected Features
1	0	100	96.97	98.44	0.97	0.984	7	*RAT*; *AAT*; *RG*; *RHE*; *RS*; *AHC*; *AVC*
2	0	96.97	100	98.44	0.97	0.984	6	*RAT*; *AAT*; *RG*; *RL*; *AIAVC*
2	1	96.43	86.11	90.63	0.83	0.906	8	*RAT*; *AAT*; *RHE*; *AHE*; *RS*; *AIAVC*; *RHC*
3	0	96.77	93.94	95.31	0.91	0.953	7	*RAT*; *AAT*; *RHE*; *RHC*; *RL*; *AVC*; *RG*
3	1	100	86.49	92.19	0.87	0.922	5	*RAT*; *AAT*; *AI*. *AHE*; *RL*
3	2	100	96.97	98.44	0.97	0.984	7	*RAT*; *AAT*; *RL*; *AIAHH*; *AVC*; *RS*
4	0	100	100	100	1	1	6	*RAT*; *AAT*; *RHE*; *AHE*; *RL*; *RG*
4	1	100	94.12	96.88	0.94	0.969	6	*RAT*; *AAT*; *RS*; *RLAI*; *RHEtp*
4	2	100	100	100	1	1	7	*RAT*; *AAT*; *RL*; *ABAVCRG*; *RHEtp*
4	3	100	96.97	98.44	0.97	0.984	6	*RAT*; *AAT*; *RL*; *AVCRG*; *R Intersection (IR)*

**Table 5:** Result of the classification with Nu-SVC for each series after the feature selection.

k	m	Sensit.	Specif.	Accur.	Youd.	ROC	#	Selected Features
1	0	96.77	93.94	95.31	0.91	0.953	7	*RAT*; *AAT*; *RS*; *RHE*; *RG*; *AHC*; *AVC*
2	0	100	100	100	1	1	6	*RAT*; *AAT*; *AI*; *AVCRG*; *RL*
2	1	96.97	100	98.44	0.97	0.984	8	*RAT*; *AAT*; *AHE*; *RHC*; *AI*; *AVC*; *RS*; *RHE*
3	0	96.97	100	98.44	0.97	0.984	7	*RAT*; *AAT*; *RHC*; *RL*; *RG*; *RHE*; *AVC*
3	1	96.77	93.94	95.31	0.91	0.953	5	*RAT*; *AAT*; *AI*; *AHE*; *RL*
3	2	100	96.97	98.44	0.97	0.984	7	*RAT*; *AAT*; *RL*; *AI*; *AHH*; *AVC*; *RS*
4	0	100	96.97	98.44	0.97	0.984	6	*RAT*; *AAT*; *RL*; *AHE*; *RG*; *RHE*
4	1	96.77	93.94	95.31	0.91	0.953	6	*RAT*; *AAT*; *RS*; *RHEtpRL*; *AI*
4	2	100	100	100	1	1	7	*RAT*; *AAT*; *RS*; *RHEtp*; *RL*; *AB*; *AVC*
4	3	100	100	100	1	1	6	*RAT*; *AAT*; *IR*; *RL*; *RG*; *AVC*

**Table 6:** Related work and their main features.

Work	Dataset Size	Acquisition Rate	Thermal Stress	Result Sens./Spec.
Anbar et al. [34]	100	100 images/s	Electric fan	95%/95%
Wishart et al. [35]	106	1.2 images/s	Cold air	70%/48%
Gerasimova et al. [36]	47	50 images/s	Uninformed	76%/86%
Saniei et al. [37]	50	Uninformed	Water with ice	86%/61%
Silva et al. [15]	70	0.066 images/s	Electric fan	98%/100%
Baffa and Lattari [38]	137	0.066 images/s	Electric fan	97%/100%
Our work	64	0.066 images/s	Electric fan	100%/100%
